# Acute Angle Closure Glaucoma Attack in a Patient With Hermansky-Pudlak Syndrome: A Case Report

**DOI:** 10.7759/cureus.68752

**Published:** 2024-09-05

**Authors:** Sebastián J Vázquez-Folch, Gabriel A Jiménez-Berríos, Natalio Izquierdo

**Affiliations:** 1 School of Medicine, Universidad Central del Caribe, Bayamón, PRI; 2 Department of Surgery, School of Medicine, University of Puerto Rico, Medical Sciences Campus, San Juan, PRI

**Keywords:** acute angle closure glaucoma, hermansky-pudlak syndrome, oculocutaneous albinism (oca), pupillary block, yag iridotomy

## Abstract

Hermansky-Pudlak syndrome (HPS) is a rare autosomal recessive disorder characterized by oculocutaneous albinism, bleeding diathesis, and ceroid deposition leading to complications such as pulmonary fibrosis and colitis. This case report describes a 63-year-old female with HPS type 1 (HPS1) who developed acute angle closure glaucoma following cataract surgery with anterior intraocular lens implantation. The patient presented with severe acute pain in the right eye, which was diagnosed as a pupillary block leading to acute angle closure glaucoma. Preoperative planning included the administration of aminocaproic acid to mitigate bleeding risk, given the patient's underlying bleeding diathesis. Yttrium aluminium garnet (YAG) laser iridotomy was performed, resulting in a significant reduction of intraocular pressure and stabilization of the patient's condition.

## Introduction

Hermansky and Pudlak [1] first described patients with a clinical triad including oculocutaneous albinism (OCA), bleeding diathesis [2], and ceroid deposition that may lead to pulmonary fibrosis [3] and/or colitis [4]. They are also prone to suffer immunodeficiencies [1]. It is estimated to affect one in 500,000-1,000,000 people.

Individuals with Hermansky-Pudlak syndrome (HPS) exhibit numerous ocular manifestations, including periodic pendular nystagmus, reduced visual acuity, refractive errors, strabismus, iris transillumination defects, foveal hypoplasia, and an albinotic mid-peripheral retina [5]. Prior research has indicated that patients with oculocutaneous albinism and HPS commonly develop early cataracts [6].

The syndrome is inherited in an autosomal recessive trait. Nonetheless, pseudodominance has been observed in areas with a high prevalence of the condition [5]. Mutations in several genes [7] have been implicated in the syndrome. Some of these genes are *HPS1*, *AP3B1*, and *HPS3*. Mutations on those genes cause HPS1, 2, and 3, respectively. In Puerto Rico, both type 1 and type 3 HPS have been identified [8].

We present a case of a patient with Hermansky-Pudlak syndrome type 1 (HPS1) who underwent cataract extraction with anterior intraocular lens (IOL) implantation and subsequently experienced an acute angle closure glaucoma episode.

## Case presentation

A 63-year-old female patient with Hermansky-Pudlak syndrome type 1 (HPS1) underwent cataract extraction and anterior intraocular lens implantation in the right eye. The patient has a gene homozygous mutation in the *HPS1* gene (c. 1472_1487dup p.His497Glnfs*90). Twenty years later, postoperatively, the patient experienced acute, severe pain in the right eye. Ocular examination revealed periodic alternating nystagmus, best corrected visual acuity of 20/400 in the right eye and 20/200 in the left eye, an anterior chamber intraocular lens in the right eye, iris bombé in the right eye, iris transillumination in both eyes, a posterior chamber intraocular lens in the left eye, foveal hypoplasia, and an albinotic mid-periphery in both eyes. Intraocular pressure measurements were 34 mm Hg in the right eye and 12 mm Hg in the left eye.

Upon diagnosing a pupillary block, a hematology consultation was sought before performing a laser iridotomy in the right eye. To mitigate the risk of iris bleeding, aminocaproic acid (500 mg) was administered in five tablet doses prior to the procedure.

**Figure 1:**
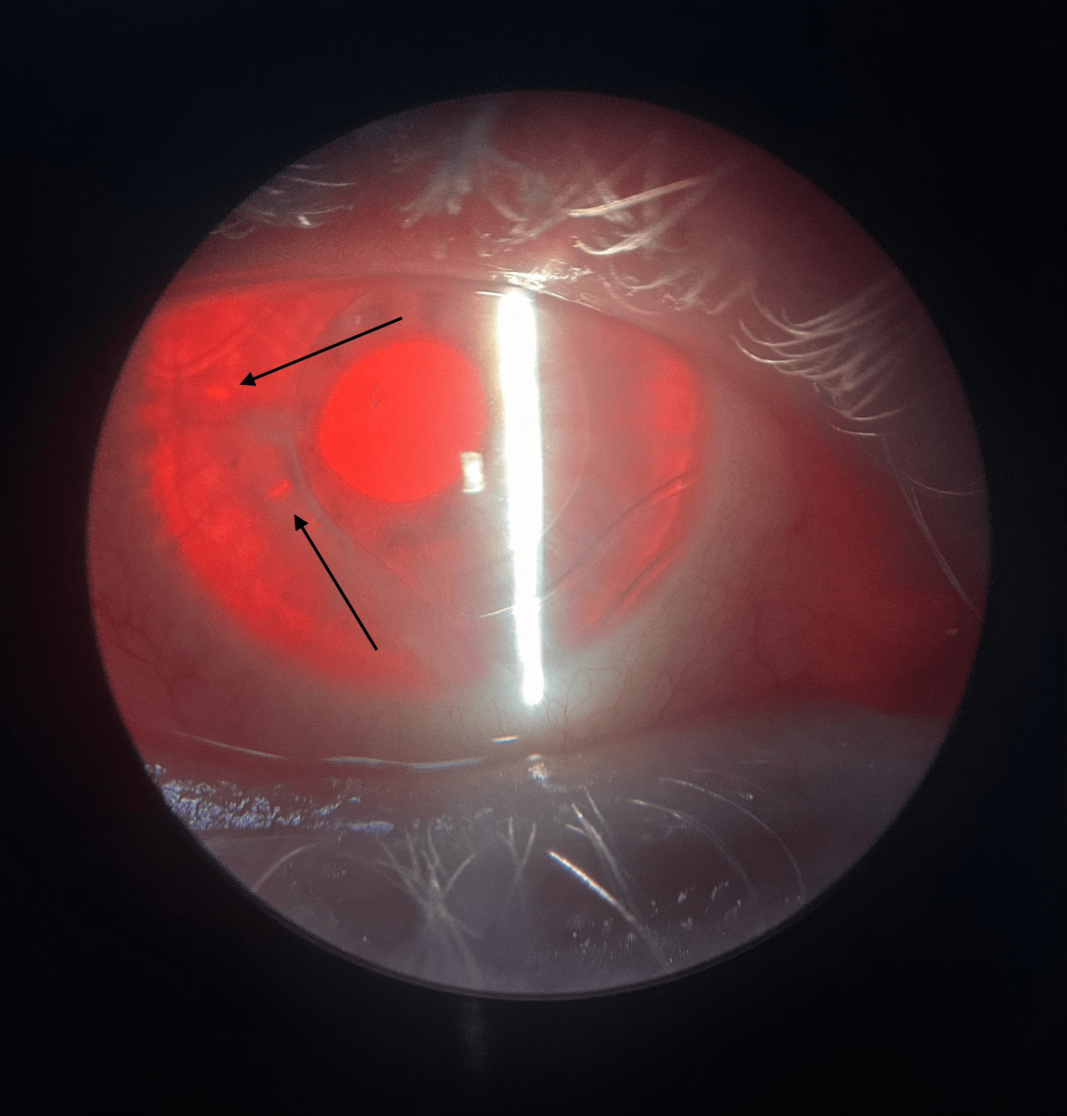
Slit lamp view of the two peripheral iridotomies. The two black arrows point to the two iridotomies.

A drop of pilocarpine 2% was instilled into the affected eye. As depicted in Figure 1, two peripheral iridotomies (PIs) were created using a yttrium aluminium garnet (YAG) laser. Post-procedure, a drop of brimonidine tartrate 0.2% and a drop of prednisolone acetate 1% were instilled. Intraocular pressure was re-evaluated 30 minutes following the procedure, with a result of 17 mm Hg. The patient was prescribed loteprednol 0.2% and brimonidine 0.2% drops for postoperative care and was advised to continue taking aminocaproic acid tablets, as advised by the hematologist.

At the follow-up visit one week later, the patient's visual acuity improved to 20/200 in both eyes. Intraocular pressure had stabilized to 19 mm Hg in the right eye and 15 mm Hg in the left eye.

## Discussion

Previous studies have reported presenile cataracts in patients with oculocutaneous albinism (OCA) [9]. Ocular manifestations in patients with HPS are similar to patients with other types of albinism [10]. In our case, the patient developed cataracts in both eyes. An anterior chamber intraocular lens was placed in the right eye, and a posterior chamber intraocular lens was implanted in the left eye. The findings in our patient are consistent with the occurrence of presenile cataracts in individuals with albinism [11]. 

Phacoemulsification and IOL have been shown to significantly improve visual acuity, reduce photophobia, and enhance the overall quality of life in patients with OCA [6]. In our case, these benefits were also observed, as the patient experienced marked improvements in visual function following bilateral cataract surgery with IOL implantation.

The patient experienced an acute angle closure glaucoma attack in the right eye after undergoing cataract surgery and anterior intraocular lens implantation. The intraocular pressure was significantly elevated in the right eye (34 mm Hg) compared to the left eye (12 mm Hg), reflecting the acute nature of the glaucoma episode. This condition was attributed to a pupillary block caused by vitreous obstruction, a complication compounded by the patient's inherent bleeding tendency associated with Hermansky-Pudlak syndrome (HPS) [2]. A study by Warren and co-workers have reported that performing laser peripheral iridotomy compared to surgical peripheral iridectomy have similar outcomes for patients requiring the procedure [12]. The study also reported that patients undergoing PI had a shorter duration of surgery. For this reason, performing a laser iridotomy in the postoperative period for patients with HPS may reduce the risk of intraocular bleeding compared to a surgical iridectomy.

Preoperative administration of aminocaproic acid was crucial to minimize the risk of iris bleeding, highlighting the necessity for meticulous preoperative planning in patients with HPS. The laser iridotomy procedure, which involved creating two peripheral iridotomies using a YAG laser, successfully reduced the intraocular pressure in the right eye from 34 mm Hg to 19 mm Hg one week postoperatively. Aminocaproic acid was also administered postoperatively, as recommended by the hematologist, to avoid secondary bleeding. This outcome underscores the efficacy of laser iridotomy in managing pupillary block glaucoma in patients with HPS.

This case highlights the importance of a multidisciplinary approach in managing patients with HPS, particularly when they present with complex ocular complications. Hematology consultation was essential in addressing the bleeding risk prior to the laser procedure. The perioperative use of medications, such as pilocarpine, brimonidine tartrate, and prednisolone acetate, was also critical in controlling intraocular pressure and inflammation.

Overall, the successful management of this patient's condition illustrates the need for comprehensive and coordinated care involving various specialties to effectively address the unique challenges posed by patients with the syndrome.

## Conclusions

In conclusion, this case of Hermansky-Pudlak syndrome (HPS) highlights the essential need for accurate diagnosis and classification of the condition. The effective management of acute angle closure glaucoma in this patient underscores the significance of individualized treatment plans tailored to the unique challenges of patients with HPS, particularly bleeding diathesis and ocular abnormalities. Additionally, performing a laser iridotomy in the postoperative period may offer a safer alternative to surgical iridectomy, particularly in reducing the risk of intraocular bleeding, which is a significant concern in patients with Hermansky-Pudlak syndrome (HPS). Future research should focus on directly comparing the outcomes of laser iridotomy versus surgical iridectomy in HPS patients, with a particular emphasis on evaluating the risk of intraocular bleeding, a complication to which these patients are particularly susceptible. This comparison could provide valuable insights into optimizing surgical strategies for managing glaucoma in this high-risk population. Administering aminocaproic acid both preoperatively and postoperatively is critical in mitigating bleeding tendencies following laser iridotomy in these patients. This case further emphasizes the importance of a multidisciplinary approach in achieving optimal outcomes for patients with rare genetic disorders.
